# Deletion of the Nuclear Localization Sequences and C-Terminus of PTHrP Impairs Embryonic Mammary Development but also Inhibits PTHrP Production

**DOI:** 10.1371/journal.pone.0090418

**Published:** 2014-05-01

**Authors:** Kata Boras-Granic, Pamela Dann, Joshua VanHouten, Andrew Karaplis, John Wysolmerski

**Affiliations:** 1 Section of Endocrinology and Metabolism, Yale University School of Medicine, New Haven, Connecticut, United States of America; 2 Lady Davis Institute for Medical Research, Jewish General Hospital, McGill University, Montreal, Canada; University of Rouen, France, France

## Abstract

Parathyroid hormone-related protein (PTHrP) can be secreted from cells and interact with its receptor, the Type 1 PTH/PTHrP Receptor (PTHR1) in an autocrine, paracrine or endocrine fashion. PTHrP can also remain inside cells and be transported into the nucleus, where its functions are unclear, although recent experiments suggest that it may broadly regulate cell survival and senescence. Disruption of either the PTHrP or PTHR1 gene results in many abnormalities including a failure of embryonic mammary gland development in mice and in humans. In order to examine the potential functions of nuclear PTHrP in the breast, we examined mammary gland development in PTHrP (1–84) knock-in mice, which express a mutant form of PTHrP that lacks the C-terminus and nuclear localization signals and which can be secreted but cannot enter the nucleus. Interestingly, we found that PTHrP (1–84) knock-in mice had defects in mammary mesenchyme differentiation and mammary duct outgrowth that were nearly identical to those previously described in PTHrP^−/−^ and PTHR1^−/−^ mice. However, the mammary buds in PTHrP (1–84) knock-in mice had severe reductions in mutant PTHrP mRNA levels, suggesting that the developmental defects were due to insufficient production of PTHrP by mammary epithelial cells and not loss of PTHrP nuclear function. Examination of the effects of nuclear PTHrP in the mammary gland *in vivo* will require the development of alternative animal models.

## Introduction

Parathyroid hormone-related protein (PTHrP) was initially discovered as a cause of hypercalcemia in patients with cancer [1], [2], [3], [4], [5]. It is encoded by a single gene that is a member of a small family that also includes the genes for parathyroid hormone (PTH) and tuberoinfundibular peptide 39 (TIP-39) [1]. PTHrP is initially translated as a 139, 141 or 173 amino acid protein that undergoes post-translational processing into a variety of smaller peptides [1], [6], [7]. The amino-terminus of PTHrP is highly homologous to PTH and the two peptides share a common G protein-coupled receptor, called the type 1 PTH/PTHrP receptor (PTHR1) [8], [9]. PTHrP is widely expressed by at least some cells in almost all organs, especially during fetal development [1], [6], [10]. A series of studies both in cells *in vitro* and in genetically engineered mice *in vivo* have demonstrated that PTHrP contributes to the regulation of cell proliferation, cell differentiation, and cell death and is required for the proper development of the skeleton, mammary gland, teeth, vascular system and pancreas [1], [11], [12], [13], [14], [15], [16], [17], [18], [19], [20]. In many developing tissues, PTHrP is expressed within epithelial cells while the PTHR1 is expressed in surrounding mesenchymal cells, suggesting a paracrine mode of action [21]. In support of this idea, the developmental defects noted in PTHrP^−/−^ embryos are generally also seen in PTHR1^−/−^ embryos [13], [14].

In addition to being secreted from cells, PTHrP can also act inside cells through an intracrine pathway. Transcription of the *PTHLH (PTHrP) gene* can be initiated from an alternative downstream start site that bypasses the signal peptide and allows PTHrP to remain in the cell [22], [23]. Alternatively, PTHrP can also bind to its receptor at the cell surface and be transported back into the cell after internalization [22]. PTHrP contains a classic nuclear localization sequence (NLS) located between amino acids 84–93 that allows it to traffic from the cytoplasm into and out of the nucleus in a regulated fashion. In cultured cells, nuclear PTHrP influences cell proliferation and/or apoptosis, and often appears to oppose the effects of secreted PTHrP. In normal vascular smooth muscle cells and in breast, colon and prostate cancer cells the nuclear pathway stimulates cell proliferation, protects cells from apoptosis or anoikis and stimulates cell migration, while secreted PTHrP inhibits cell proliferation and promotes cell death [12], [24], [25], [26], [27]. In order to examine the consequences of loss of nuclear PTHrP *in vivo*, two groups recently replaced the endogenous mouse *Pthlh gene* with truncated versions encoding forms of mutant PTHrP (PTHrP 1–66 or PTHrP 1–84) that exclude the NLS and C-terminus [28], [29]. In both cases, exclusion of PTHrP from the nucleus resulted in growth failure, premature osteoporosis, reduced hematopoesis, altered energy metabolism and, ultimately, premature death at about 2 weeks of age. There was a decrease in the proliferation of chondrocytes, osteoblasts, neurons, and bone marrow cells, and an increase in apoptosis or senescence at these sites as well as in the thymus and spleen. Loss of mid-region and C-terminal PTHrP was associated with increased expression of senescence markers such as p21 and p16^INK4a^ and decreased expression and nuclear trafficking of Bmi-1, which is involved in stem/progenitor cell maintenance [29], [30]. This phenotype suggests that nuclear PTHrP may participate broadly in the regulation of cell proliferation and survival as well as stem/progenitor cell maintenance or self-renewal.

PTHrP and the PTHR1 are both required for the formation of the breast. In human and mouse embryos, the mammary gland initially forms as an epidermal placode that invaginates into the underlying dermis to form a bud-like structure that becomes surrounded by 3–4 concentric layers of specialized mammary mesenchyme cells [31], [32], [33]. Around embryonic day 16 (E16) in mice, the mammary bud initiates a process of branching ductal morphogenesis forming a rudimentary duct system that grows out of the mammary mesenchyme into a second stromal compartment known as the mammary fat pad. The initiation of mammary ductal outgrowth is triggered by signals from the mammary mesenchyme, which also signals the overlying epidermis to form the specialized skin of the nipple [31], [33], [34]. By birth the neonatal mammary gland consists of a simple duct system characterized by a single primary duct and approximately 10–15 initial branches. Disruption of PTHrP to PTHR1 signaling in either mice or humans causes defects in mammary mesenchyme development and leads to the failure of subsequent morphogenesis [19], [20], [34]. PTHrP is produced by mammary epithelial cells beginning at the placode stage and the PTHR1 is widely expressed by the underlying dermal mesenchyme. As the mammary bud invaginates, PTHrP induces the proliferation and differentiation of the mesenchymal cells at least in part by activating BMP and Wnt signaling cascades [35], [36], [37]. In the absence of a normal mammary mesenchyme, the epithelial buds from PTHrP^−/−^ and PTHR1^−/−^ embryos either give rise to a severely stunted duct system or fail to form any ducts. In addition, the mammary epithelial progenitor cells within the bud differentiate into skin cells [34], [38]. Finally, the mammary mesenchyme fails to induce the formation of the nipple. These studies have underscored the importance of the mammary mesenchyme in embryonic mammary development and have demonstrated that paracrine interactions between PTHrP and the PTHR1 are required for mammary ductal outgrowth, nipple formation and the maintenance of the embryonic mammary stem/progenitor cells [34].

Our presumption has been that the actions of PTHrP on embryonic mammary development require it to be secreted from epithelial cells and to activate classical cell surface PTHR1 signaling on mesenchymal cells. However, Toribio and colleagues described defects in embryonic mammary development in the PTHrP (1–66) knock-in mice suggesting that the nuclear pathway might also contribute to PTHrP’s actions on this organ [29]. Specifically, they reported that while the development of the initial duct system appeared normal, nipple formation was absent, implicating defects in the mammary mesenchyme. The report describing the PTHrP (1–84) knock-in mouse by Miao et al did not specifically examine mammary development [39]. However, we reasoned that if nuclear PTHrP signaling was important to breast development, then any defects in the embryonic mammary gland should be similar in both PTHrP (1–66) knock-in mice and PTHrP (1–84) knock-in mice. Therefore, we performed a detailed analysis of embryonic mammary development in PTHrP(1–84) knock-in embryos and found that they had defects in mammary outgrowth similar to those previously reported in PTHrP^−/−^ and PTHR1^−/−^ embryos. However, we also found that these mice produced very little PTHrP in their mammary buds, making it difficult to discern a specific contribution of nuclear PTHrP to embryonic mammary development.

## Materials and Methods

### Ethics Statement

All animal experiments were reviewed and approved by the Yale Institutional Animal Care and Use Committee.

### Animals

PTHrP(1–84) knock-in mice were obtained from Dr. Andrew Karaplis (McGill University, Montreal, Canada) and have been described previously [39]. They were maintained on a C57/Bl6 background and were genotyped as described [39]. PTHrP^−/−^ mice and controls were maintained on a CD-1 background and were identified as described previously [13], [20]. PTHR1^−/−^ mice were maintained on a Black Swiss background and were genotyped as previously reported [14], [38].

### Histology and Immunohistochemistry

Mammary gland whole mounts were prepared from neonatal mice using standard techniques. Whole mice were fixed for 12–16 hours in 4% paraformaldehyde. The ventral skin was then dissected and stained with carmine-aluminum as previously described [20]. After staining, the mammary glands and adjacent skin was cleared and mounted on a microscope slide, examined and photographed using a stereomicroscope. Histological sections of embryonic mammary glands were prepared as previously detailed [20]. After fixation in 4% paraformaldehyde, strips of ventral skin containing the mammary buds were embedded on edge in paraffin. Serial 5–7 micron sections were cut and mammary buds were identified by examining the unstained sections. Pertinent slides were then either stained with hematoxylin and eosin using standard conditions or were used for immunohistochemistry or TUNEL assay as described below.

Ductal outgrowth was measured in millimeters as the distance from the origin of the main duct at the nipple/skin interface to the end of the longest duct. These measurements were performed on whole mounts of neonatal mammary glands as described above. The analysis was based on 12 mammary glands from 3 WT mice and 15 mammary glands from 4 PTHrP(1–84) knock-in mice. Averages and SEM’s as well as statistical significance using the unpaired t-test were calculated using Prism 6.00 for Windows (Graph Pad Software, La Jolla, CA).

Immunohistochemistry was performed on paraffin sections of embryonic mammary buds using standard techniques as previously reported [36]. Primary antibodies included SC-56836 against the estrogen receptor (Santa Cruz Biotechnology, Santa Cruz, CA), PG-21 directed against the androgen receptor (Millipore, Billerica, MA), C12A5 directed against Lef-1 (Cell Signaling Technologies, Danvers, MA), Ab#558686 against GATA3 (BD Pharmingen-BD Biosciences, San Jose, CA) or anti-tenascin C (a kind gif from Dr. T. Yoshida, Mie University School of Medicine, Japan). Staining was detected using Vector Elite ABC kits (Vector Laboratories, Burlingame, CA) and 3,3′-diaminobenzidine (Vector Laboratories) as a chromagen or Alexa Fluor 488-conjugated goat-anti-mouse, secondary antibody (Life Technologies, Grand Island, NY) for immunofluorescence. For WT embryos, 5 buds from 2 embryos were stained for ER, 3 buds from 2 embryos were stained for AR, 9 buds from 2 embryos were stained for tenascin C, 10 buds from 3 embryos were stained for Lef1. For PTHrP(1–84) knock-in embryos, 10 buds from 3 embryos were stained for ER, 5 buds from 3 embryos were stained for AR, 10 buds from 5 embryos were stained for tenascin C and 5 buds from 2 embryos were stained for Lef1. For PTHrP^−/−^ embryos, 4 buds from 2 embryos were stained for ER, 3 buds from 2 embryos were stained for AR, 12 buds from 2 embryos were stained for tenascin C and 4 buds from 2 embryos were stained for Lef1. For PTHR^−/−^ embryos, 2 buds from 1 embryo were stained for ER, 2 buds from 1 embryo were stained for AR, 3 buds from 2 embryos were stained for tenascin C and 4 buds from 1 embryo were stained for Lef1. Representative buds are shown in the figures.

Mammary buds were isolated from E14 male embryos of the various genotypes as previously described [40]. Paraffin sections of the appropriate buds were assayed for histology and apoptosis using the terminal deoxynucleotidyl transferase-mediated dUTP nick end-labeling (TUNEL) method as determined with the Roche Fluorescein Kit (11684795910, Roche, Indianapolis, IN, USA). The histological assessment of mammary buds in male embryos was based on the examination of 6 buds from 2 different WT embryos and 12 buds from 2 different PTHrP(1–84) knock-in embryos. The TUNEL analysis was based on 6 buds from 1 WT embryo and 6 buds from 1 PTHrP(1–84) knock-in embryo.

#### Quantitative RT-PCR

RNA was prepared from mammary buds as previously reported [37]. Briefly, E13.5 mammary buds were dissected from freshly harvested, unfixed embryos of the desired genotypes that were placed in saline at 4°C. Two independent batches of RNA were prepared from between 15–22 mammary buds harvested from 3 embryos of each genotype. Freshly dissected buds were trimmed of excess skin and placed into RNAlater (Qiagen, Germantown, MD) prior to RNA preparation. RNA samples were isolated using Qiagen RNeasy MicroPlus Kit (Qiagen) as per the manufacturers instructions. RNA integrity was analyzed using an Agilent 2100 Bioanalyzer and RNA quality measures (RIN scores) were greater than 9.0. Approximately 100 to 150 ng RNA was amplified with NuGEN amplification (NuGEN Technologies Inc., San Carlos, California, USA) before performing QRT-PCR. For QRT-PCR, 100 ng of RNA was analyzed in 20 µl reactions using the EXPRESS One-Step SuperScript qRT-PCR Kit (Life Technologies, Grand Island, NY) in an iQ5 thermal cycler (BioRad, Hercules, CA). Each reaction was performed in triplicate. TaqMan Gene Expression Assays (Life Technologies) were used to measure expression levels of PTHrP (*Pthlh*; assay Mm00436057_m1), and *Gata3* (Life Technologies, assay Mm00484683_m1). The PTHrP (*Pthlh*) primers amplify nucleotides between 573–688 (RefSeq NM_008970.3), which are shared between PTHrP (1–84) mRNA and wild-type PTHrP mRNA. PTHrP expression was determined using the 2^−ΔΔCT^ method in the 6 RNA samples for each genotype (triplicate reactions from each of 2 independent pools of WT, PTHrP (1–84) knock-in, or PTHrP^−/−^ mammary buds). Values were represented as mean +/− SEM relative to the WT expression level, which was arbitrarily set to 100%. The one-sample t-test (Prism 6.00 for Windows, Graph Pad Software, La Jolla, CA) was used to test whether the mean levels of PTHrP expression in PTHrP(1–84) knock-in and PTHrP^−/−^ mammary buds differed significantly from its expression level (set to 1) in WT mammary buds.

## Results

### Mammary Ductal Outgrowth is Stunted in PTHrP(1–84) Knock-In Embryos

Miao et al knocked a mutant form of PTHrP containing a premature termination codon at amino acid 85 into the murine *Pthlh* locus, resulting in the production of PTHrP(1–84), which lacks the NLS and C-terminal portions of PTHrP abrogating its localization to the nucleus [39]. Loss of the NLS and C-terminal portions of PTHrP in this model caused severe growth restriction, multiple organ abnormalities and premature death characterized by widespread cellular senescence. Given the suggestion from these studies that PTHrP might contribute to stem cell maintenance and given the recent suggestions that stem cells within the embryonic mammary gland contribute to ductal development [41], [42], [43], we attempted to clarify the role of nuclear PTHrP in embryonic/neonatal mammary development by examining the mammary glands from PTHrP (1–84) knock-in mice.

**Figure 1:**
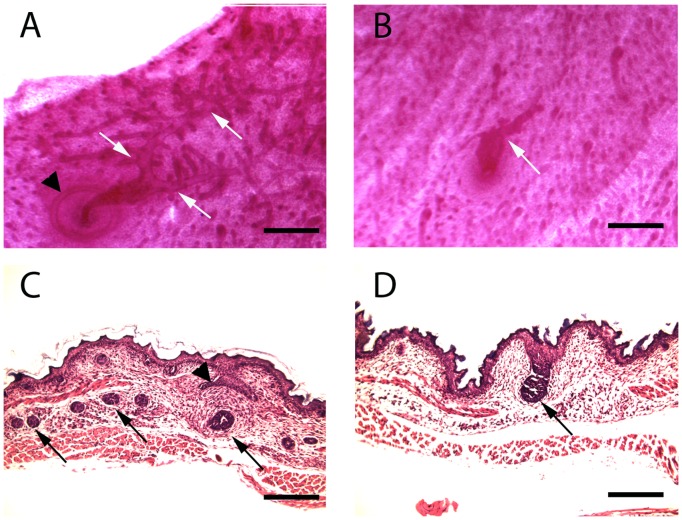
Abnormal ductal outgrowth from PTHrP (1–84) knock-in embryonic mammary buds. A&B. Typical whole mounts of mammary epithelial ducts from WT and PTHrP (1–84) knock-in female mice on day 2 of life. In the WT mammary gland (A), the primary duct arises from the skin and gives rise to a series of branching ductal structures (white arrows). The nipple sheath (black arrowhead) can be seen as a concentric circle around the origin of the primary duct from the skin. In the PTHrP (1–84) knock-in mouse (B), the primary duct is short and dilated and gives rise to very stunted outgrowths (white arrow). In addition, there is no nipple structure around the origin of the abnormal primary duct. Scale bars in A&B represent 0.6 mm. C&D. Histological sections through the mammary glands of female WT and PTHrP (1–84) knock-in embryos on E18. In WT embryos (C), the nipple sheath (arrowhead) is a crescent-shaped structure that protrudes from the epidermis into the dermis around the origin of the primary duct. Cross sections of the mammary ducts are highlighted with arrows. Note that the duct has grown away from the nipple and down into the sub-dermis. (D) shows a stunted mammary structure from a PTHrP (1–84) knock-in embryo. Note that it still resembles a bud and that there are no mammary ducts in the sub-dermis. Scale bars in C&D represent 800 microns.

**Figure 2:**
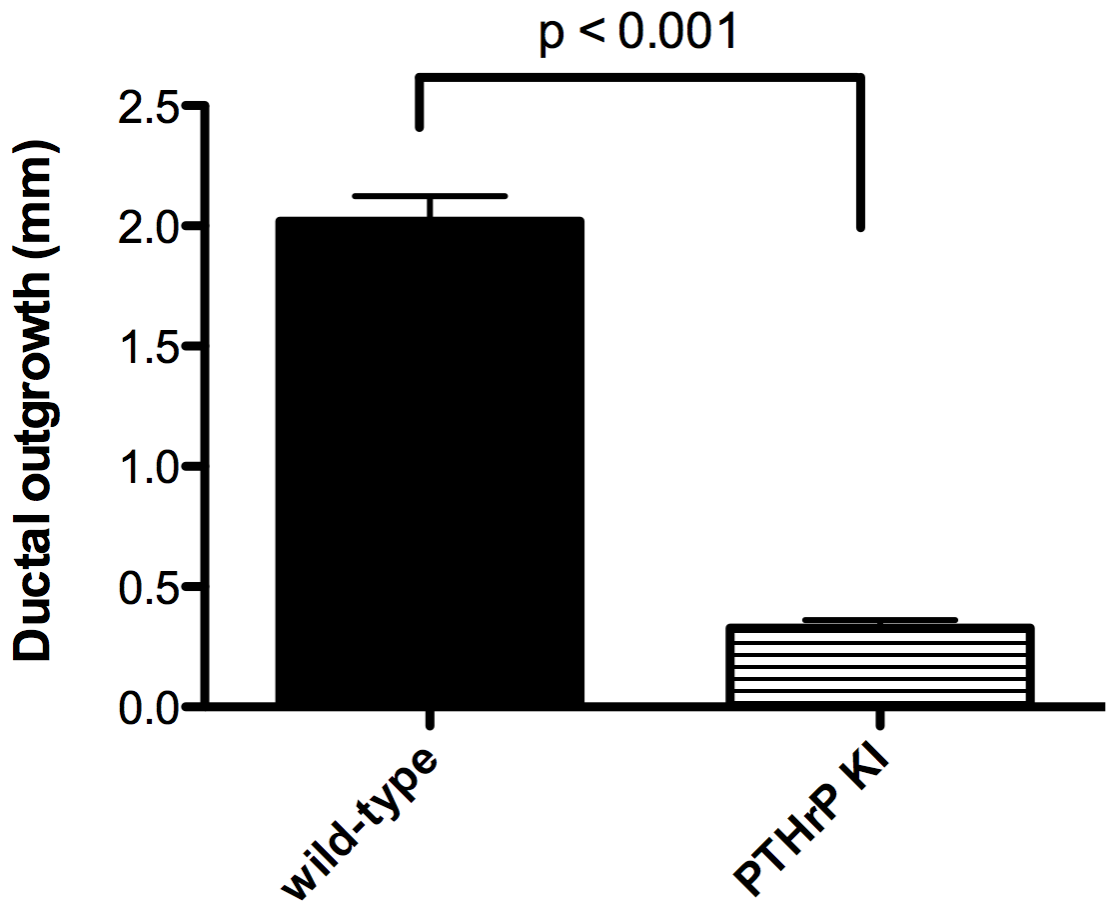
Quantitation of mammary ductal outgrowth in wild-type and PTHrP(1–84) knock-in neonatal mice. Bars represent the average ± SEM of the furthest extension of the mammary ducts away from their origin at the nipple**.** Analysis included 12 WT mammary glands from 3 separate 2-day-old females and 15 PTHrP(1–84) knock-in mammary glands from 4 separate 2-day-old mice.

In normal mouse embryos, the outgrowth of the primary duct from the mammary bud and the formation of the nipple sheath are initiated at E16. By E18, the formation of secondary branches has begun and, at birth, the gland consists of a rudimentary duct system with 10–15 branches. Therefore, we examined the ductal tree in PTHrP (1–84) knock-in mice and controls at E18 and neonatal day 2 (Figure. 1). As shown, on whole mount analysis, the neonatal WT gland consists of a primary duct that gives rise to a series of initial branches. In addition, the nipple sheath can clearly be seen as a ring of thickened epidermis surrounding the origin of the primary duct from the embryonic skin. In contrast, the glands from the knock-in mice consist of a severely stunted primary duct and one or two short, aberrant branches (Figure 1B). Furthermore, there is no nipple sheath surrounding the primary duct. As shown in Figure. 2, WT ducts had extended 2 mm on average from the nipple while ducts in PTHrP (1–84) knock-in embryos had only grown 0.3 mm. On histological examination of the WT gland on E18, the nipple sheath can be seen as invaginations of epidermis on either side of the primary duct, which can be seen penetrating into the nascent mammary fat pad forming within the sub-dermis (Figure. 1C). In contrast, in the knock-in embryo, there is no nipple sheath and the stunted duct has not penetrated out of the dermis and into the mammary fat pad (Figure. 1D).

### PTHrP(1–84) Knock-In Embryos Display Defects in Mammary Mesenchyme Formation

**Figure 3:**
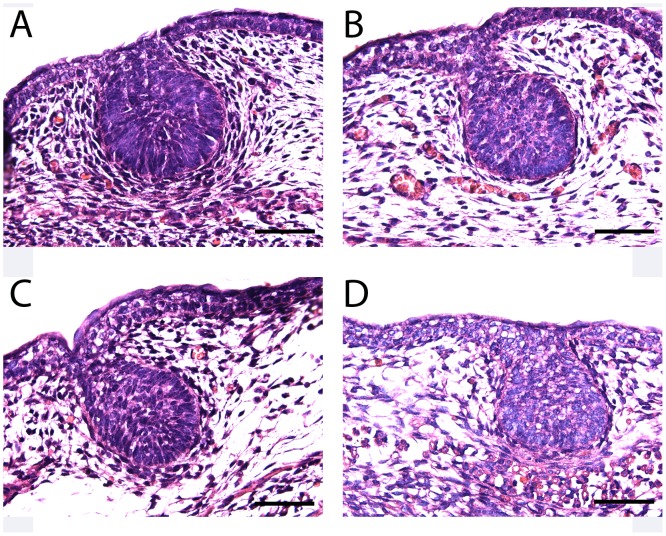
Histology of PTHrP (1–84) knock-in embryonic mammary buds. Embryonic mammary buds from female embryos on E15 from the following genotypes: WT (A), PTHrP (1–84) knock-in (B), PTHrP^−/−^ (C), PTHR1^−/−^ (D). Note the elongated and condensed mesenchymal cells arrayed in a concentric fashion around the epithelial bud in the WT embryo (A). In all three mutant strains (B–D), the epithelial cells appear normal but there appear to be fewer layers of mesenchymal cells around the buds and they are less elongated and not organized in a concentric orientation. Scale bars in all panels represent 200 microns.

Outgrowth of the embryonic duct system requires signals from the specialized mesenchyme that surrounds the epithelial bud [31], [32], [34]. Previous experiments have demonstrated that the formation and differentiation of the mammary mesenchyme depends on PTHrP, secreted from the mammary epithelial bud, interacting with the PTHR1 on surrounding mesenchymal cells [20], [34], [35], [38], [44]. Therefore the failure of proper mammary ductal outgrowth in PTHrP^−/−^ and PTHR1^−/−^ embryos has been interpreted to result from a breakdown in critical mesenchymal to epithelial signaling due to the lack of paracrine PTHrP to PTHR1 signaling. Given the similarity between the defective ductal outgrowth observed in PTHrP (1–84) knock-in as compared to PTHrP^−/−^ and PTHR1^−/−^ embryos, we next examined mammary mesenchyme development at E15. As shown in Figure 3, in E15 WT female embryos, the mammary buds consist of a sphere of mammary epithelial cells suspended from the epidermis by a narrower neck of epithelial cells all surrounded by 3–4 layers of elongated compacted fibroblast-like cells arrayed in a concentric fashion around the epithelial bud and also located between the epidermis and the bud neck. These mesenchymal cells are also associated with increased amounts of eosinophilic extracellular matrix as compared with the general dermal mesenchyme (Figure 3A). In the PTHrP(1–84) knock-in buds (Figure 3B), the epithelial structure is similar to the WT. However, there are fewer surrounding mesenchymal cells, which are only loosely arrayed in a concentric orientation and have reduced amounts of associated extracellular matrix. As can be seen in Figures 3C&D, the histological findings in the PTHrP(1–84) knock-in buds are similar to those in PTHrP^−/−^ (Figure 3C) and PTHR1^−/−^ (Figure 3D) mammary buds.

**Figure 4:**
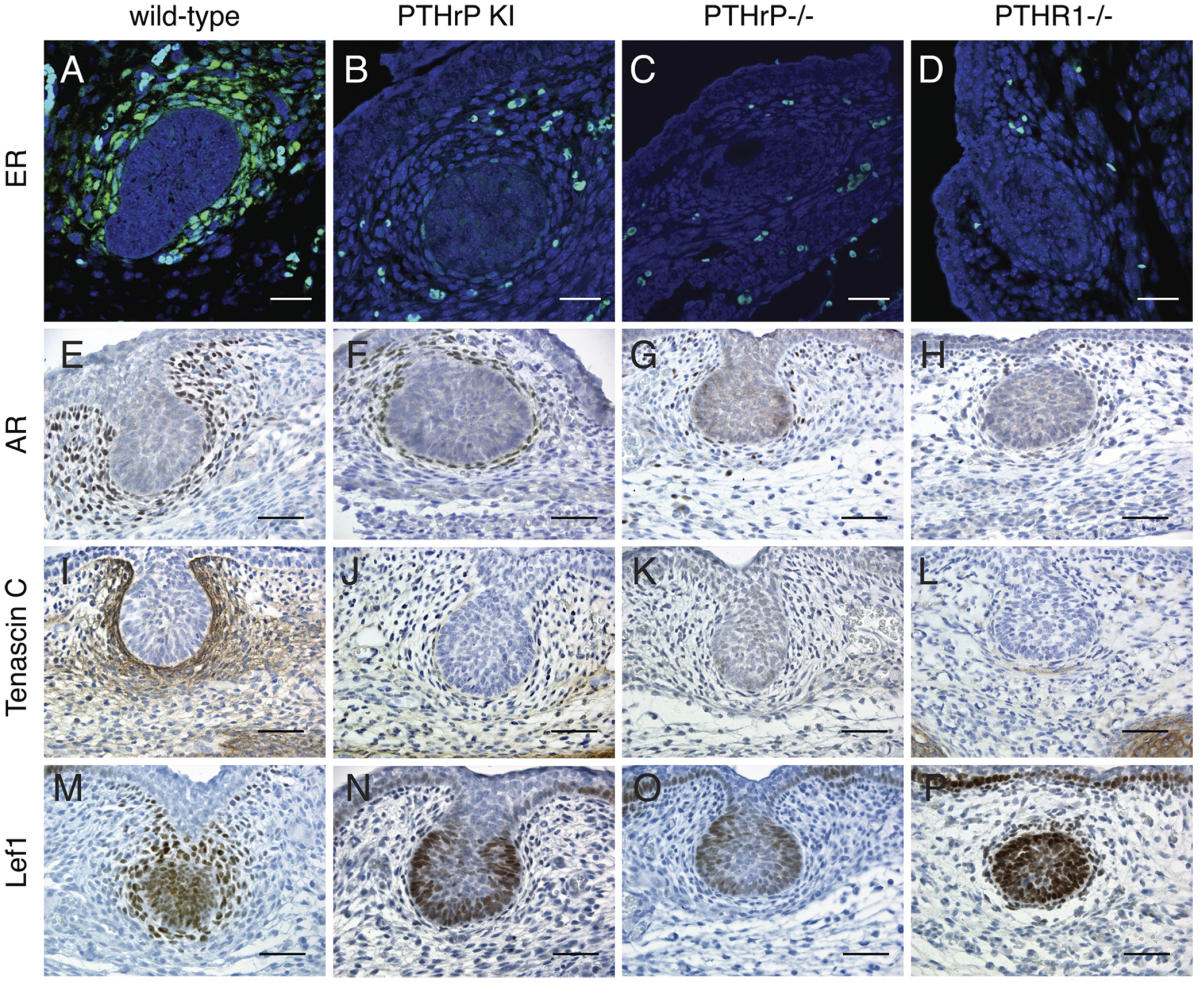
Loss of mammary mesenchyme markers in PTHrP (1–84) knock-in mammary buds. Sections from female E15 mammary buds were stained for estrogen receptor (ER, A–D), androgen receptor (AR, E–H), tenascin C (I–L) and Lef1 (M–P). In WT buds (A, E, I, M) ER, AR and tenascin C are strongly expressed in the mammary mesenchyme around the epithelial buds, while Lef1 is expressed in both the epithelial and mesenchymal cells in the bud. Note the reduction in staining for these markers in the mammary mesenchyme of the PTHrP (1–84) knock-in buds (B, F, J, N). These findings are similar to although not as severe as the loss of these markers from the mammary mesenchyme in buds from PTHrP ^−/−^ (C, G, K, O) and PTHR1^−/−^ (D, H, L, P) embryos. Scale bars represent 200 microns.

We next examined a series of molecular markers characteristically expressed by the specialized mammary mesenchyme (Figure 4). As expected, the estrogen receptor (ER), the androgen receptor (AR), and tenascin C are all expressed within the WT mammary mesenchyme but not the surrounding dermal mesenchyme. We used ER staining to quantify the numbers of mammary mesenchyme cells surrounding the sections of epithelial buds. In WT controls, bud sections contained 68±11 ER-positive cells within 3–4 layers. In addition, Lef1 is expressed within the WT mammary mesenchyme and the WT mammary epithelial cells, but not in those basal keratinocytes destined to contribute to the nipple. In contrast, in the PTHrP(1–84) knock-in buds, the expression of all the markers were greatly reduced in the mammary mesenchyme and Lef1 was abnormally expressed in the basal keratinocytes in the neck of the bud and by keratinocytes immediately adjacent to the mammary epithelium. Sections of PTHrP(1–84) knock-in buds were surrounded by 8±2 ER-positive cells and no bud had even one complete layer of mammary mesenchyme. In comparison, the PTHrP^−/−^ and PTHR^−/−^ buds displayed an absence of mammary mesenchyme markers and upon quantitation had no surrounding ER-positive cells. These results suggest that mammary mesenchyme differentiation is severely impaired in PTHrP(1–84) knock-in embryos although not completely absent as in the PTHrP^−/−^ and PTHR1^−/−^ embryos.

### Loss of Sexual Dimorphism during the Development of the PTHrP(1–84) Knock-In Mammary Buds

**Figure 5:**
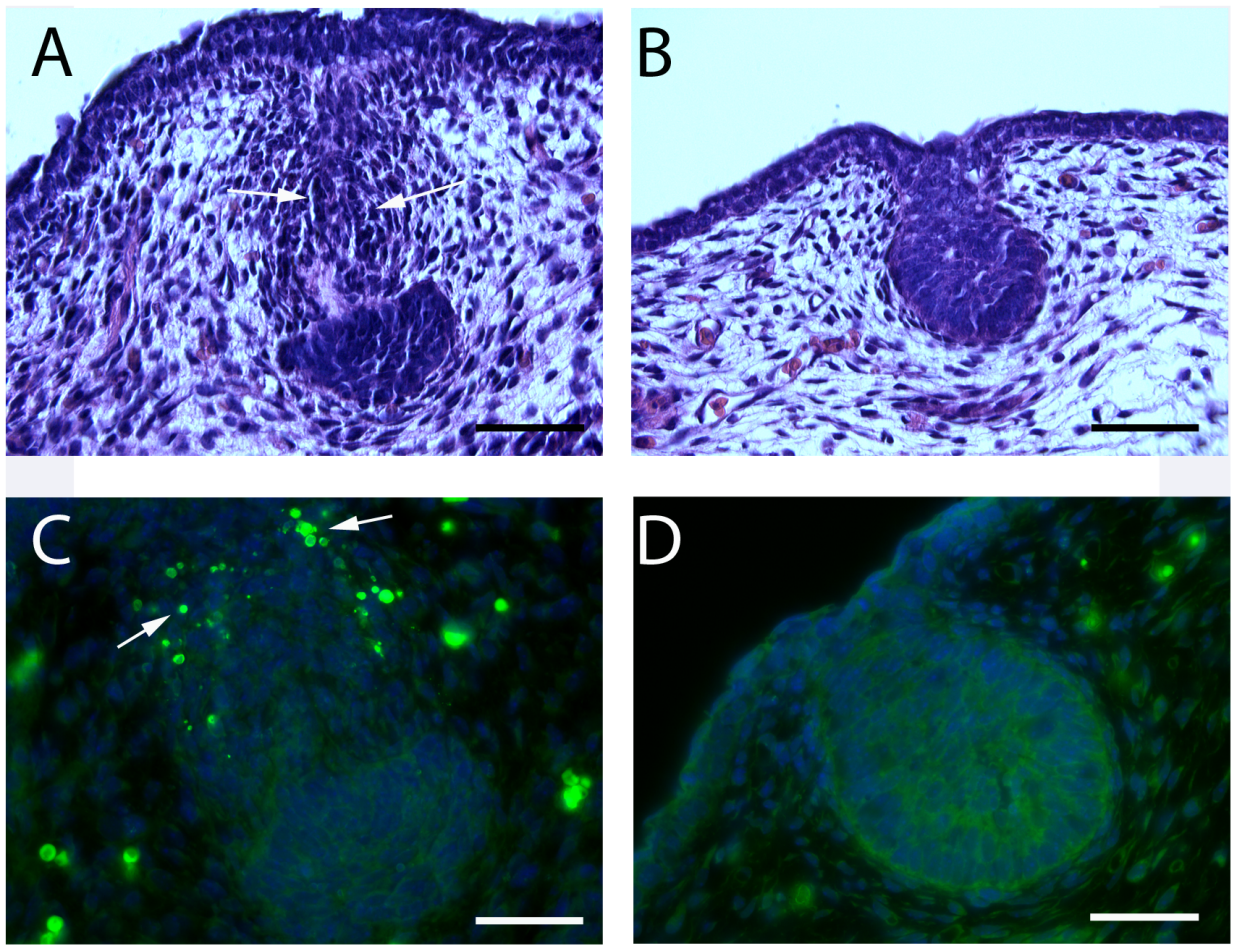
Failure of sexual dimorphism in PTHrP (1–84) knock-in mammary buds. A&B show H&E-stained sections through mammary buds harvested from male embryos on E14. The WT (A) bud shows the typical androgen-driven condensation of the mesenchymal cells and constriction of the neck of the mammary bud (white arrows). This is associated with severing of the connection between the epidermis and the bud and the distortion of the epithelial portion of the mammary bud. In contrast, this response is absent in the mammary buds from PTHrP (1–84) knock-in male embryos, which look exactly like the female knock-in mammary buds (see Fig. 3B). C&D show TUNNEL staining of male E14 mammary buds. As expected, many of the condensed mesenchymal cells in the WT bud (C) are undergoing apoptosis (white arrows) in response to endogenous androgens. However, there are no TUNEL-positive cells within the mammary buds in male PTHrP (1–84) knock-in embryos (D) on E14. Scale bars represent 250 microns in A&B and 440 microns in C&D.

In wild-type male mice, embryonic mammary gland development is disrupted between E14 and E15 by circulating androgens that interact with the androgen receptor expressed within the mammary mesenchyme [31], [32]. Androgens cause the mesenchymal cells to condense further and constrict around the neck of the epithelial bud to sever its connection to the epidermis. In addition, androgens cause widespread apoptosis of the mesenchymal cells as well as variable numbers of epithelial cells [40]. The process results either in the absence of the mammary epithelial duct system or in the retention of a very rudimentary duct that is disconnected from the skin and unable to grow out into the mammary fat pad. Defects in mammary mesenchyme differentiation in PTHrP^−/−^ and PTHR1^−/−^ embryos results in a failure of this androgen mediated destruction of the mammary bud and the loss of sexual dimorphism in mammary bud development [40]. Given the similarities between the defects in the PTHrP(1–84) knock-in, PTHrP^−/−^ and PTHR1^−/−^ mammary mesenchyme, we next assessed mammary bud development in male PTHrP(1–84) knock-in embryos on E14. As expected, the WT male embryos showed evidence of mesenchymal condensation and thinning or disruption of the stalk of the mammary bud (Figure 5A). Furthermore, TUNEL staining demonstrated widespread apoptosis of the WT mammary mesenchyme cells (Figure 5C). In contrast, on E14, the mammary buds from male PTHrP(1–84) knock-in embryos resembled the normal mammary buds of female embryos. There was no condensation of mesenchyme around the mammary stalk and no apoptosis occurred within these cells (Figures 5B&D). These findings are similar to those previously reported for mammary buds from male PTHrP^−/−^ and PTHR1^−/−^ embryos [40].

### PTHrP(1–84) Knock-In Mammary Buds have Greatly Diminished PTHrP Expression

**Figure 6:**
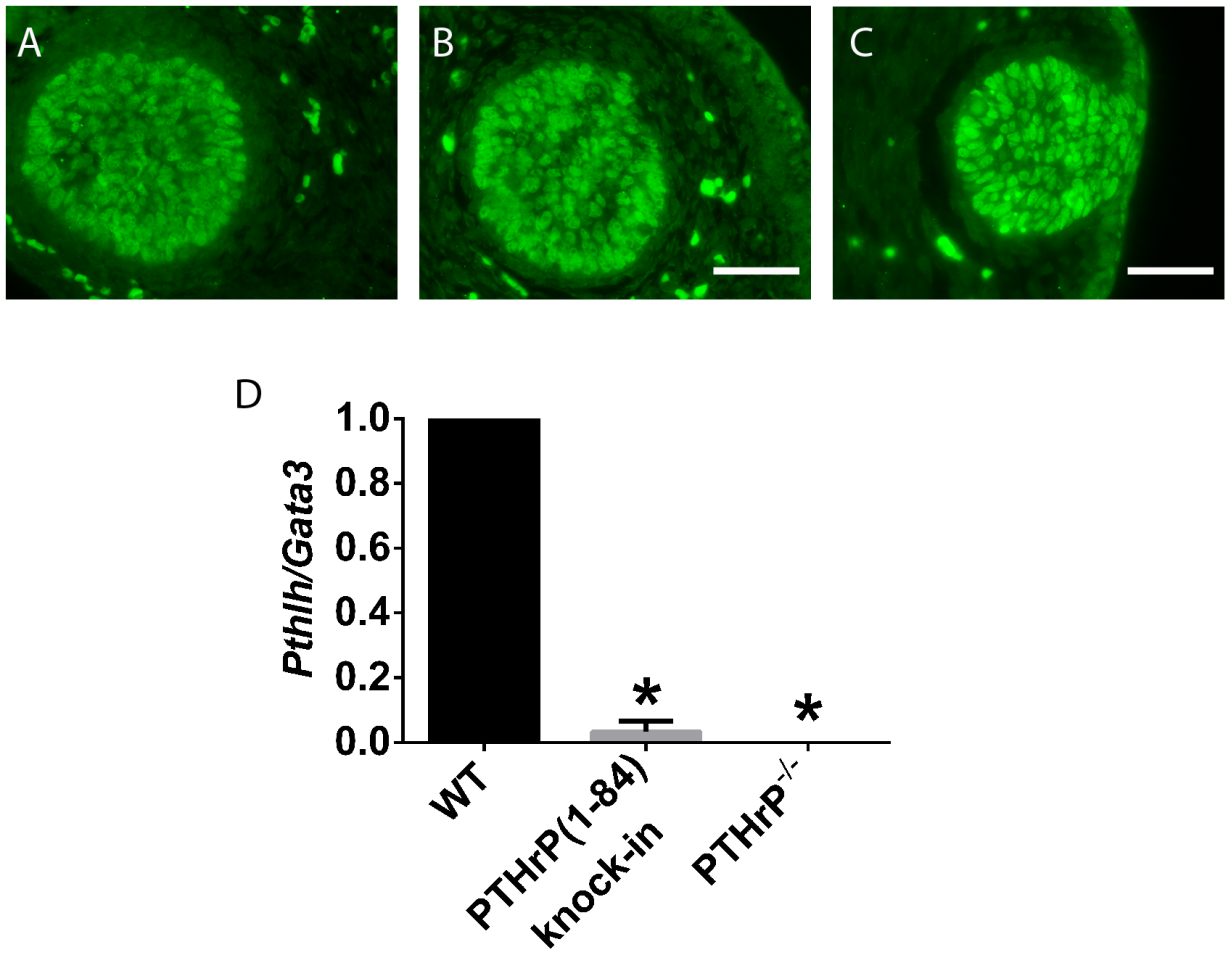
PTHrP mRNA levels are reduced in PTHrP (1–84) knock-in mammary buds. A–C. GATA3 staining of mammary buds at E15 taken from WT (A), PTHrP(1–84) knock-in (B), and PTHrP^−/−^ (C) embryos. GATA3 expression is comparable in the epithelial cells of the different types of mammary buds. Scale bars represents 440 microns. D. PTHrP (*Pthlh)* mRNA levels were measured in mammary buds isolated from WT, PTHrP (1–84) knock-in and PTHrP^−/−^ embryos on E13 and normalized to epithelial cell content by correcting for *Gata3* mRNA expression. *Pthlh gene* expression is severely reduced in the PTHrP (1–84) knock-in buds as compared to WT buds and almost as low as in PTHrP^−/−^ mammary buds. *denotes statistically significant difference compared to WT. The p value for the comparison between WT and PTHrP (1–84) knock-in buds was 0.02. The p value for the comparison between WT and PTHrP^−/−^ buds was <0.001.

Our expectation was that excluding PTHrP from the nucleus would cause cell autonomous defects. Therefore, given that PTHrP is produced by mammary epithelial cells and that the PTHR1 is expressed on mesenchymal cells, we found it curious that mammary buds in the PTHrP(1–84) knock-in embryos reproduced the defects in mammary mesenchyme cells that occur in both the PTHrP^−/−^ and PTHR1^−/−^ mammary buds. The simplest explanation for these observations was that knocking the mutant PTHrP(1–84) construct into the *Pthlh gene* locus somehow compromised PTHrP production by embryonic mammary epithelial cells. Therefore, we microdissected mammary buds from WT, PTHrP(1–84) knock-in, and PTHrP^−/−^ embryos on E13 (before circulating androgens affect the WT buds) and performed QPCR to quantify *Pthlh mRNA* levels using primers that would recognize both *WT Pthlh mRNA* and *Pthlh (1–84) mRNA*. In order to correct for any variations in epithelial cell content among the different pools of mammary buds we normalized *Pthlh* mRNA expression to *Gata3* mRNA expression, which is expressed specifically by mammary epithelial cells. This analysis is shown in Figure 6. First, as seen in Figures 6 A–C, GATA3 immunostaining was the same in WT, PTHrP(1–84) knock-in and PTHrP^−/−^ epithelial buds. As demonstrated in Figure 6D, in PTHrP(1–84) knock-in buds, *Pthlh mRNA* levels, normalized for *Gata3* mRNA expression, were reduced to almost the same background levels as measured in the PTHrP^−/−^ buds. Therefore, it is likely that very little PTHrP is produced by the knock-in epithelial cells.

## Discussion

In this report, we document that PTHrP (1–84) knock-in embryos have defects in mammary mesenchyme differentiation almost identical to those previously documented in PTHrP^−/−^ and PTHR1^−/−^ embryos. In the PTHrP^−/−^ and PTHR1^−/−^ strains, the mammary epithelial buds form but are surrounded by fewer layers of mesenchyme that is poorly organized and that fails to express molecular markers typical of the normal condensed mammary mesenchyme in wild-type controls. In addition, sexual dimorphism in bud development is lost; ductal outgrowth is severely stunted; and the nipple sheath fails to form, all events that depend on cues from the mesenchymal cells. We observe essentially the same phenotype in the PTHrP(1–84) knock-in mammary buds. Although there remains some minimal residual mammary mesenchyme around the knock-in buds, it is clearly below the threshold required to direct proper morphogenesis of the duct and nipple. However, we also document severely reduced *Pthlh mRNA* levels in mammary buds harvested from PTHrP (1–84) knock-in embryos, suggesting that the defects in mammary development in these mice may result from severely reduced levels of PTHrP and not just the loss of nuclear PTHrP.

A series of observations suggests that PTHrP regulates embryonic mammary development by being secreted and activating the PTHR1 on neighboring cells. First, PTHrP expression is limited to the epithelial cells in the embryonic mammary buds in both mouse embryos and human fetuses, while the *PTHR1 gene* is expressed by the surrounding mesenchymal cells in both species [19], [20], [34], [45]. Second, loss of PTHR1 on mesenchymal cells in PTHR1^−/−^ buds produces defects in mammary development identical to those caused by the loss of PTHrP from epithelial cells in PTHrP^−/−^ buds [20], [34], [38], [40]. Likewise, human fetuses with Bloomstrand chondrodysplasia and homozygous null mutations in the *PTHR1 gene* lack breast duct development similar to PTHrP^−/−^ mice [19]. Third, transplantation experiments using different recombinations between epithelium and mesenchyme from PTHR1^−/−^ and WT mammary buds demonstrated that PTHR1^−/−^ epithelium was capable of normal outgrowth when paired with WT mesenchyme, but that PTHR1^−/−^ mesenchyme was not able to support the outgrowth of WT epithelium [44]. These results rule out the possibility of any low-level, cell-autonomous effects of the PTHR1 in epithelial cells and also demonstrate that cell-autonomous effects of PTHrP in the epithelium are insufficient to support normal development. Fourth, transgenic expression of PTHrP specifically in mammary epithelial cells rescues mammary gland development in PTHrP^−/−^ embryos [20]. Finally, treatment with amino-terminal PTHrP(1–36) rescues the outgrowth of PTHrP^−/−^ mammary buds in organ culture demonstrating that the NLS and C-terminal regions are not required to mediate the actions of PTHrP on mammary development [35]. In light of these data, it was difficult for us to envision how loss of nuclear PTHrP trafficking in the epithelial cells where the *PTHrP gene* is expressed could cause defects in mammary mesenchyme differentiation and function, processes that require expression of the PTHR1 on the mesenchymal cells. Clearly, the simplest interpretation of our current findings in the PTHrP (1–84) knock-in embryos is that the epithelial cells produce too little PTHrP to provide sufficient activation of the PTHR1 on the neighboring mesenchymal cells.

We were surprised that mammary buds harvested from PTHrP (1–84) knock-in embryos demonstrated such low levels of *Pthlh mRNA* expression given that Miao and colleagues had demonstrated appropriate PTHrP (1–84) expression in embryonic fibroblasts cultured from PTHrP (1–84) knock-in embryos [28]. In addition, at birth, PTHrP (1–84) knock-in mice lacked developmental abnormalities, such as a failure of tooth eruption and severe shortening of limbs, that are characteristic of loss of PTHrP signaling in PTHrP^−/−^ and PTHR1^−/−^ mice [13], [14], [28]. Wild-type *Pthlh mRNA* is labile and normally found at low levels in most cells [1], [6]. However, the embryonic mammary buds represent some of the most prominent foci of *Pthlh mRNA* expression and we speculate that this level of expression may depend on regulatory elements that increase transcription rates or alter the half-life of the message and that were disrupted by deleting portions of the mid-region and C-terminal sequences of the *Pthlh gene*. If this is the case, similar issues may complicate interpretation of the mammary phenotype in the PTHrP (1–66) knock-in model. Turibio and colleagues validated expression of PTHrP (1–66) in skin, respiratory epithelium and cultured embryonic fibroblasts using immunohistochemistry, immunoblotting or immunoassay [29]. However, they did not specifically examine PTHrP expression in mammary buds and given the more extensive PTHrP deletion introduced into this mouse model, it is likely that mRNA levels are reduced in mammary epithelial cells. We did not evaluate other tissues in our study, but our findings clearly caution that proper levels of PTHrP production must be carefully validated in each cell type of interest when using these mouse models. They also suggest that there may be interesting regulatory elements within the deleted portions of PTHrP that control its mRNA expression and/or half-life in a cell-type specific manner.

In closing, our studies do not rule out the possibility that nuclear PTHrP participates in the regulation of mammary development, physiology or disease. In fact, intriguing studies in cell lines suggest that nuclear PTHrP may contribute to the malignant behavior of breast cancer cells [46], [47], [48]. We remain intrigued by the overall findings in the PTHrP (1–84) knock-in and PTHrP (1–66) knock-in models, and, especially, the suggestion that nuclear PTHrP might be important to the regulation of stem/progenitor cells in a variety of tissues including the mammary gland [29], [39]. However, this study demonstrates that the current mouse models will not allow detailed study of these issues in mammary epithelial cells *in vivo*. The development of more finely targeted mutations interrupting nuclear PTHrP trafficking in a mammary specific manner will be required to address these important questions.
